# Resistance integrons: class 1, 2 and 3 integrons

**DOI:** 10.1186/s12941-015-0100-6

**Published:** 2015-10-20

**Authors:** Yang Deng, Xuerui Bao, Lili Ji, Lei Chen, Junyan Liu, Jian Miao, Dingqiang Chen, Huawei Bian, Yanmei Li, Guangchao Yu

**Affiliations:** College of Light Industry and Food Sciences, South China University of Technology, Guangzhou, 510640 China; Institute of Agro-products Processing, Anhui Academy of Agricultural Sciences, Hefei, 230031 China; Department of Laboratory Medicine, First Affiliated Hospital of Guangzhou Medical College, Guangzhou, 510120 China; The Third Affiliated Hospital of Sun Yat-sen University, Guangzhou, 510630 China; Guangzhou Women and Children’s Medical Center, 9 Jinsui Road, Guangzhou, 510620 China; First Affiliated Hospital of Jinan University, Guangzhou, 510620 China

**Keywords:** Antimicrobial resistance, Mobile genetic elements, Horizontal transfer, Resistance integrons

## Abstract

As recently indiscriminate abuse of existing antibiotics in both clinical and veterinary treatment leads to proliferation of antibiotic resistance in microbes and poses a dilemma for the future treatment of such bacterial infection, antimicrobial resistance has been considered to be one of the currently leading concerns in global public health, and reported to widely spread and extended to a large variety of microorganisms. In China, as one of the currently worst areas for antibiotics abuse, the annual prescription of antibiotics, including both clinical and veterinary treatment, has approaching 140 gram per person and been roughly estimated to be 10 times higher than that in the United Kingdom, which is considered to be a potential area for the emergence of “Super Bugs”. Based on the integrons surveillance in Guangzhou, China in the past decade, this review thus aimed at summarizing the role of integrons in the perspective of both clinical setting and environment, with the focus on the occurrence and prevalence of class 1, 2 and 3 integrons.

## Background

Antibiotics, as compounds or substances that kill or inhibit the growth of microorganisms, have been regarded as one of the greatest contributions to medicine and humanity in the 20th century and used to treat a wide range of infectious diseases caused by bacteria, for both animals and human beings [1–4]. However, as recently indiscriminate abuse of existing antibiotics in both clinical and veterinary treatment leads to proliferation of antibiotic resistance in microbes and poses a dilemma for the future treatment of such bacterial infection, antimicrobial resistance has been considered to be one of the currently leading concerns in global public health, and reported to widely spread and extended to a large variety of microorganisms, which will consequently result in an increasing number of clinical failures in bacterial mediated diseases [2, 3, 5]. A number of resistance mechanisms are responsible for the emergence and prevalence of antimicrobial resistance, and such mechanisms have been divided into genetic mutation occurred at a low frequency and acquisition of various genes mediated resistance to their host microorganisms. As consequence, acquisition of resistance genes has been regarded as major contributor for the wide distribution and spread of antimicrobial resistance, via either vertical transfer and horizontal transfer, with the latter mechanism involving mobile genetic elements such as plasmids and transposons [3]. As mostly carried by plasmids or contained within a transposon, integrons as well as its mechanism and role played in the distribution of microorganisms have been well established and documented [6, 7], which had also been considered to contribute to the unleashing of “Super Bugs” [3, 8]. In China, as one of the currently worst areas for antibiotics abuse, the annual prescription of antibiotics, including both clinical and veterinary treatment, has approaching 140 gram per person and been roughly estimated to be 10 times higher than that in United Kingdom [3, 8, 9].

Since the first report in 1989 [10], the molecular mechanism and mobility of integrons, including the excision and integration for gene cassettes, had been investigated and validated for the following years [6, 7, 11]. Moreover, its occurrence in clinical microorganisms and its role played in antimicrobial resistance were also widely studied for the past decades [12, 13].

## Structure

**Fig. 1:**
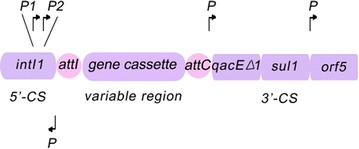
Schematic representation of a class 1 integron. *P1* promoter for transcription of gene cassettes, *P2* second promoter that is usually inactive, *int* integrase gene, *attI1* integration site, *qacE* partially deleted gene that encodes quaternary ammonium compound resistance, *sulI* sulphonamide resistance, *orf5* unknown function, *P* promoters of the *qacE*△ and *sulI* genes, *attC* sequence on the gene cassette recognized by the integrase

**Fig. 2:**
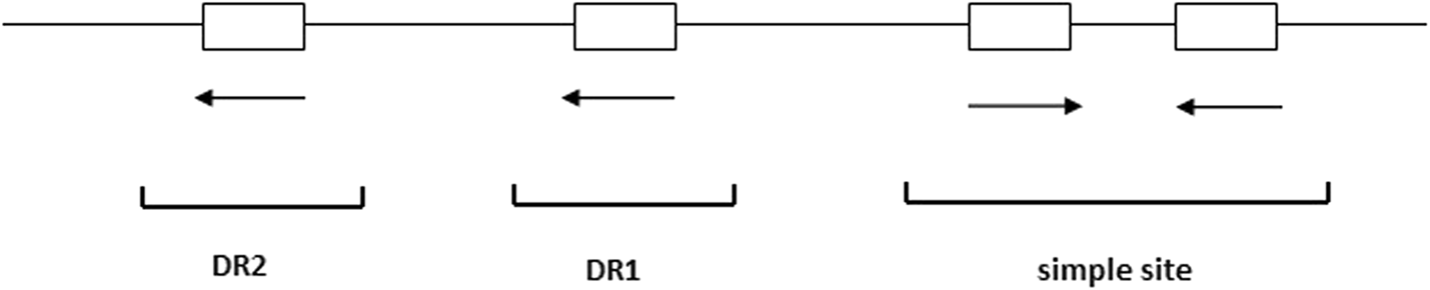
Structure of the *attI1* site. The essential part of the *attI1* site is formed by the simple site, the strong binding site and the intervening sequence. For details see Hall et al. [7]

An integron is generally defined by the presence of an integrase gene (*intI*) and a proximal primary recombination site (*attI*) (Fig. 1) [2, 14]. The amino acid sequences of IntI integrases have been used as a basis for dividing integrons into ‘classes’, with those carrying *intI1* defined as ‘class 1’, *intI2* as ‘class 2’, *intI3* as ‘class 3’, etc. *intI1*, *intI2* and *intI3* were first identified in association with mobile genetic elements and *intI4* and others with chromosomal integrons. As the most commonly selected target for the detection of an integron, *intI* encodes an integrase (IntI) of the tyrosine recombinase family, which is characterized by the distinct presence of invariant RHRY (with Y being the catalytic tyrosine) amino-acids in the conserved motifs called box 1 and box 2 that discriminate *intI* within the other XerC-related integrase) [2]. IntI-catalysed recombination between *attI* and/or *attC* sites results in insertion or excision of cassettes (Fig. 1). The class 1 integrase (IntI1) recognises three types of recombination site: *attI1*, *attC* and secondary sites. Binding domains and consensus sequences have been determined for these. The *attI1* site is a simple site which contains two inverted sequences that bind the integrase, and two additional integrase-binding sites known as strong (DR1) and weak (DR2) (Fig. 2) [11, 15].

### The attC sites

The *attC* region contains two simple sites, each composed of a pair of conserved ‘core sites’ (7 or 8 bp), referred to as R″ and R′, L′ and L″ [2]. The R′ and R″ sites are part of the RH consensus sequence, which is more or less equivalent to the RH simple site. The L′ and L″ sites are part of the LH consensus sequence, which is more or less equivalent to the LH simple site [8, 11]. The LH and RH sites in the attC are possibly distinguished by the integrase, which might explain the orientation of integration of the gene cassettes. L″ also appears to be significant for orientation [15]. The LH simple site is not only required for orientation but also enhances RH activity [16, 17]. The *attC* sites are generally associated with a single ORF in a structure termed gene cassettes, which are not necessarily observed in integrations, but once integrated they become part of the integron [11].

### Gene cassettes

According to previous reports, cassettes located within the variable region of integrons are sometimes absent in the structure of integrons [18]. Via specific excision and integration, gene cassettes are integrated between two recombination sites (*attI* and *attC*) and thus become part of the integron, and exist in either the independent circular DNA molecule which is unable for stably maintain during cell division or the linear form which is created by a highly orientation-specific insertion of the free circular element into the integron [2]. Despite possession of a coding sequence, gene cassettes are generally found to be lack of promoters to constitute the mobile component of the system, and most cassettes encode resistance against antibiotics cover a wide range of antibiotics, with up to date more than 130 distinct antibiotic resistance genes characterized via unique *attC* sites [19, 20]. Together, these cassettes confer resistance to most classes of antibiotics containing all known β-lactams, all aminoglycosides, chloramphenicol, streptothricin, trimethoprim, rifampin, erythromycin, quinolones, fosfomycin, lincomycin, and antiseptics of the quaternary ammonium-compound family [21, 22].

## Mobility

Reported as widely spread and distributed in clinical organisms, the mobility of integrons has been considered to be a major concern of clinically antibiotic resistance, which is defined as being associated with mobile DNA elements (transposons or plasmids) and antibiotic-resistance genes in addition to having a small array size and substantial heterogeneity in the sequence of *attC* sites [7, 13]. Despite the defectivity of self-transposition, currently existent integrons (mostly class 1 integron) has been considered to be a potentially mobile genetic element and commonly found to be located on plasmids as facilitation of conjugative-mediated transfer, as it contains gene cassettes that are mobile and capable of transferring to other integrons or to secondary sites in the bacterial genome. The integron system is a natural capture system and assembly platform, which allows microorganisms to incorporate gene cassettes and further convert them to functional proteins via correct expression. Each unique ORF is conceivably capable of being structured as a novel type of gene cassette and vital to decipher the mechanism governing cassette genesis. As a consequence, with the naturally huge pool of gene cassettes, integron may have the potentially limitless capacity to exchange and stockpile functional gene cassettes which consequently permits rapid adaptation to selective pressure and may ultimately endow increased fitness and advantage to the host [7, 13]. In addition, mobile genetic elements, including conjugative plasmids, transposons, insertion sequences and genomic islands, may potentially be the vast reservoirs and massive genetic pool for integron, which will further be shared among bacteria [20, 23]. With mobility from gene cassettes, integrons play key role in the dissemination and spread of resistance genes, responsible for both spread and exchange of resistance genes to a wide range of distinct antibiotics among diverse bacteria [23, 24]. Aside from clinical perspectives, a large number of reports on integrons from environmental microorganisms, as well as the high sequence diversity observed and various functional products other than resistance encoded by such cassettes, strongly indicates integrons are ancient genetic element within the genomes and may have played a critical role in evolution and adaptation for a considerable period [25].

## Classification

From the differences and divergence in the sequences of *intI*, integrons have been classified and divided into several classes. Up to date, 4 general classes of integrons have been identified and distinguished, termed classes 1–4 integrons. Known as multi-resistant integron (RIs), classes 1–3 integrons are capable of acquiring same gene cassettes via similar recombination platform, which had been supported by the in vitro excision and integration occurred via recombination sites from such integrons [11]. Most of the currently available studies on integrons had been conducted on class 1 integron, with focus on Gram-negative microorganisms. As a distinct type of integron, class 4 integron was firstly identified on the small chromosome of *Vibriocholerae* and found to be an integral component of many γ-proteobacterial genomes [26, 27], which had also been considered to be a leading concern on both antimicrobial resistance and bacterial genome evolution, despite the limitation of the associated reports within the species of *Vibrio*. The remaining classes of integrons may also contain antibiotic resistance gene cassettes, but their worldwide prevalence remains low [28].

### Class 1 integron

**Fig. 3:**
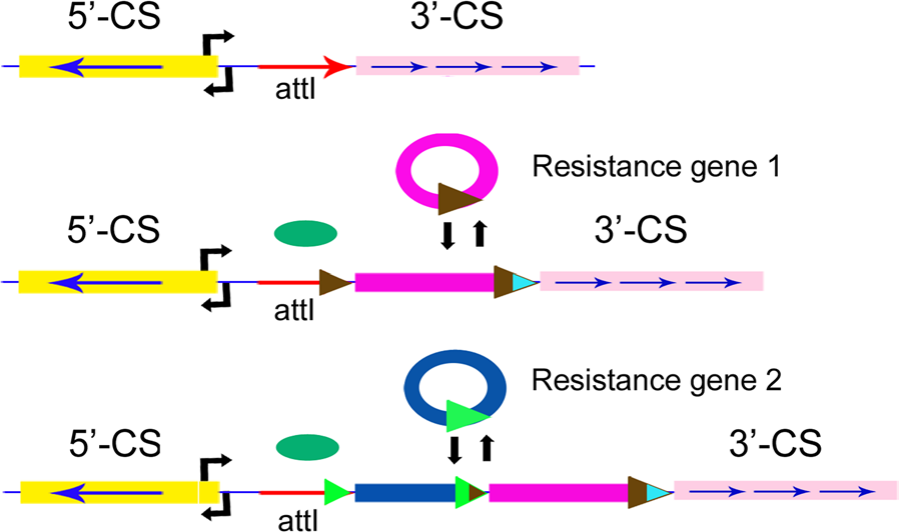
Integration and excision of gene cassettes by site-specific recombination. Intl encoded by the *intl* gene in the integron catalyses recombination between the *attl1* site of the integron and/or the attC site(s) of gene cassette(s) resulting in insertion or excision of a cassette. One or more noncassette resistance genes may be inserted at the position of the 3′-CS. *Horizontal arrows* indicate the opposite orientations of *intI* and cassette-borne genes

Integrons have been found in approximately 9 % of the sequenced bacterial genomes, and class 1 integron platform is the most ubiquitous and has been the most commonly reported among clinical bacteria and remains the focus of numerous studies [29, 30]. Considered to be directly linked with Tn*402*-like transposons and associated with Tn*3* transposon family (Tn*21* or Tn*1696*), class 1 integron is not self-movable, while other mobile genetic elements such as conjugative plasmids and transposons associated are able to serve as vehicles for the intraspecies and interspecies transmission of genetic material through site-specific recombination reaction mediated by either the Tn*21* integrase or the integron integrase *IntI1* when the integration sites conform to the consensus sequence GWTMW or GNT (Fig. 3), respectively [20, 31]. Three types of recombination sites (*attI1*, *attC* and secondary sites) are able to be recognized by *intI1*, though with different recombination efficiency as recombination event between *attI1* site and *attC* has been shown slightly more efficient than recombination between two *attC* sites, and that between two *attI1* sites far less efficient, with recombination by secondary sites with *attC* more efficient than that with *attI*. As a consequence, this class of integron is capable of capturing gene cassettes via this site-specific recombination platform, and gene cassettes are also able to be further expressed from a common promoter located in the 5′-conserved segment (5′-CS) region where two potential promoter sites Pc (also known as P_ANT_) and P2 locate, with Pc approximately 200 bp upstream of the integration site [25]. Despite the dispensability for the site-specific recombination platform, Pc plays a key role in the functioning of integron as it ensures the correct expression of gene cassette, as comparatively P2 is inactive as the replacement of the optimal 17 nucleotides between the −35 and −10 boxes to only 14 nucleotides [25]. Downstream of gene cassette within a typical class 1 integron, the 3′-conserved segment (3′ CS) possesses the genes *qacE∆1* and *sul1*, encoding resistance to quaternary ammonium salts and sulfonamide, respectively [31]. *Escherichia*, *Pseudomonas*, *Salmonella*, *Staphylococcus*, *Enterococcus* and *Vibrio* have still been considered to be frequent pathogens responsible for various bacterial infections and diseases [2, 3, 32–36], and their relevant chemotherapy are clinically significant. As a common contributor to the wide distribution and spread of antimicrobial resistance, class 1 integron has been studied in various microorganisms, with its occurrence and prevalence commonly reported to be ranging from 22 to 59 % and identified in clinical Gram-negative bacteria, including *Acinetobacter*, *Aeromonas*, *Alcaligenes*, *Burkholderia*, *Campylobacter*, *Citrobacter*, *Enterobacter*, *Escherichia*, *Klebsiella*, *Mycobacterium*, *Providencia*, *Pseudomonas*, *Salmonella*, *Serratia*, *Shigella*, *Stenotrophomonas*, *and Vibrio* (Table 1) [3, 14, 16–20, 23, 32–48]. As the local studied area was concerned, class 1 integrons were commonly found in Gram-negative bacteria isolated in Guangzhou, southern China during 2001–2006 [2, 49], with an occurrence of 73.6 % (243/330), with high prevalence for *E.coli*, *K. pneumoniae*, *Acinetobacter spp.* and *Enterobacter cloacae*, except for *P. aeruginosa* (45.8 %, 54/118).

**Table 1:** Occurrence and prevalence of class 1 integron in Gram-negative microorganisms

Date	Bacterial	Occurrence of class 1 integron and the array of gene cassettes	Sampling	References
2006	*Shigella*	*EstX-aadA1* (3.85 %, 1/26)	Hiroshima prefecture, Japan; 2000–2004	[34]
2002	*Salmonella*	36.2 % (34/94); *aadA2-bla* (*PSE-1*) (61.76 % 21/34); *aadA1-aadA2-bla (PSE-1)* (38.23 %,13/34)	Animals, Japan	[33]
2000	*V. cholerae*	44/176; *aadB-aadA2-blaP1-dfrA1-dfrA15*	Thailand	[39]
2002	*Burkholderia*	29.4 % (5/17); *oxa-aac* (6′-1a)	Ireland	[38]
2004	*Campylobacter*	62/378	Ireland	[37]
2008	*Enterobacteriace*ae	50/226	Addenbrooke’s Hospital	[62]
2005	*Escherichia coli*	4/32 (12.5 %); *sat-1*-*aadA*	Meat and meat products, Norwey	[42]
2008	*E. coli*	59.5 % (355/597)	South Thailand	[65]
2011	*E. coli*		Preliminary study in Guangzhou, China	[3]
2009	*P. aeruginosa*	45.8 % (54/118)	Preliminary study in Guangzhou, China	[19]
2008	*Serratia*	1/30; *aacC1-ORFX-ORFY-aadA1*	Canada	[17]
2004	*Stenotrophomonas maltophilia*	22 % (20/93)	Kaohsiung Medical University	[36]
2013	*P. aeruginosa*	43 % 37/182	Guilan, Iran	[44]
2011	*K. pneumoniae*	18/26	Blood stream infections	[2]
2013	*S. enteritidis*	11.9 % (59)	Taiwan	[41]
2013	*S. panama*	40.0 % (20)	Taiwan	[41]
2010	*P. aeruginosa*	High prevalence	Iran	[40]
2009	*Aeromonas*	16/41 (39.02 %); *dfrA15-cmlA4-aadA2*	Hidalgo, Mexico	[32]

Class 1 integron has been well established and documented in Gram-negative microorganisms, with its role in the distribution and spread of antimicrobial resistance also verified and identified. Class 1 integrons are associated with a variety of resistance gene cassettes, but most integrons contain an *aadA* resistance determinant, encoding streptomycin-spectinomycin resistance. Trimethoprim resistance determinants are also detected frequently [12, 21, 22]. This is not surprising because trimethoprim + sulphamethoxazole has been a therapeutic combination used frequently [12]. Class 1 integrons isolated from bacteria involved in infections of man frequently also harbor gene cassettes encoding β-lactam resistance [22]. In addition, new gene cassettes encoding resistance against these aminoglycosides have been discovered during the last few years [45]. However, such studies have been significantly restricted to species of Gram-negative bacteria, with only a few examples amongst Gram-positive organisms. Up to date, class 1 integrons have been reported on Gram-positive bacteria including *Corynebacterium*, *Streptococcus*, *Enterococcus*, *Staphylococcus*, *Aerococcus* and *Brevibacterium*, and gene cassettes *aadA* and *dfrA* were most frequently detected (Table 2). In 1998, the first evidence of class 1 integron among Gram-positive bacteria was reported as the complete class 1 integron was detected on a 29-kb plasmid pCG4 associated streptomycin/spectinomycin resistance determinant from *Corynebacterium glutamicum* [50]. In 1999, *aadA* (an integron-related gene) was recovered in *E. faecalis* strain W4470, with the transfer of class 1 integron via a plasmid between *E. faecalis* of the horizontal transfer [51]. In 2002, an intI1-like gene truncated by *IS6100* was found on a 27.8-kb R-plasmid pTET3 in *C. glutamicum* LP-6, which mediated resistance to streptomycin, spectinomycin and tetracycline [23]. During 2001–2004, a total of 15 enterococcal strains isolated in Guangzhou, China were detected to be positive for class 1 integrase and 3′-conserved region of *qacE*∆*1*-*sul1*, with class 2 integrons also discovered in two *E. faecalis* strains [52]. During 2001–2002, class 1 integrons had been detected in four consecutive *Streptococcus* strains sampled from First Affiliation Hospital of Jinan University in Guangzhou, China, with an array of *dfrA12*-*orfF*-*aadA2* [52]. In 2004, class 1 integrons were recovered from several species of *Corynebacterium* spp., (*C. ammoniagenes*, *C. casei* and *C. glutamicum*), *Aerococcus* spp., *Staphylococcus* and *Brevibacterium thiogenitalis* from poultry litter [43]. As *Staphylococcus* strains are considered to be the top three contaminating pathogens (with HBV and HIV) [51], the finding of class 1 integrons in this genus from the recent decade is notable. During 2001-2006, class 1 integrons were commonly found in clinical *Staphylococcus* isolated from FAHJU and Guangdong Provincial People’s Hospital in Guangzhou, China. Within this integron investigation conducted in Guangzhou, class 1 integrons were detected in 122 MRS strains (from 262 MRS isolates, with 209 MRSA and 53 MRCNS); no class 2 or 3 integrons were obtained [2, 3, 49, 53]. In 2009, class 1 integron was identified from one *S. epidermidis* strain isolated from Bogota, Colombia, which carried the 78 % homologous *int1* and the cassette arrays of *aac6* (aminoglycoside acetylation) with resistance to aminoglycoside and *aac6′*-*aph2′* with resistance to β-lactams [54]. In 2013, class 1 integrons was reported on 81 *Staphylococcus* isolates (40.5 %, 81/200) recovered from nasal and throat swabs in Sanandaj Hospital, Iran, including 37 (40.1 %) *S. aureus*, 35 (23.5 %) *S. epidermidis* and 9 (36.0 %) *S. saprophyticus* strains [55].

**Table 2:** Occurrence and prevalence of class 1 integron in Gram-positive microorganisms

Date	Bacterial	History	Description	Ref
1998	*Corynebacterium glutamicum*	First class 1 integron evidence in G^+^ microorganisms	InCg on pCG4 (29-kb), identical to InC on pSA1700	[42]
1999	*Enterococcus faecalis*	First class 1 integron report within *Enterococcus* and the species of *E. faecalis*	*aadA* was found via hotizontal transfer between *E. faecalis*	[43]
2002	*Corynebacterium glutamicum*		On pTET-3 (27.8-kb), with a novel *aadA9* detected	
2001–2004	*Enterococcus faecalis*	First class 2 integron evidence in G^+^ microorganisms and first report of class 1 integron on the species of *E. Faecium*	Three arrays (*dfrA12*-*orfF*-*aadA*2*, dfrA17*-*aadA5* and *aadA2*) for class 1 integron and array *dfrA1*-*sat1*-*aadA1* for class 2 integron	[44]
	*Enterococcus faecium*			
2001–2002	*Streptococcus*	First class 1 integron report within *Streptococcus*	Array of *dfrA12*-*orfF*-*aadA2* detected	[44]
2004	*Corynebacterium ammoniagenes*	First class 1 integron report on *Aerococcus* spp. and the species of, *C. ammoniagenes*, *C. casei*, *Brevibacterium thiogenitalis*; also the first identification of class 1 integron from envirnmental G^+^ microorganisms	Such integrons carried various antibiotic resistance genes and may serve as the potentially large reservior of class 1 integron	[36]
	*Corynebacterium casei*			
	*Corynebacterium glutamicum*			
	*Aerococcus*			
	*Brevibacterium thiogenitalis*			
	*Staphylococcus*			
2001–2006	*Staphylococcus aureus*	First class 1 integron evidence from clinical G^+^ microorganisms from a large scale and a long study duration; first class 1 integron identification from clinical *S. aureus* and the species of *S. epidermidis*, *S. haemolyticus*, *S. hominis* and *S. warneri*	Typically class 1 integrons were observed, with *intI1* and 3′CS of ∆*qacE*, a *sulI* gene and ORF5. Four arrays (*dfrA12*-*orfF*-*aadA*2*, dfrA17*-*aadA5, aacA4*-*cmlA1* and *aadA2*) detected	[3]
	*Staphylococcus epidermidis*			
	*Staphylococcus haemolyticus*			
	*Staphylococcus hominis*			
	*Staphylococcus warneri*			
2009	*Staphylococcus epidermidis*		With 78 % homologous *int1* and the cassette arrays of *aac6*	[45]
2013	*Staphylococcus aureus*	First class 1 integron report on the species of *S. saprophyticus*	With identification rate of class 1 integron as 40.5 % (81/200)	[46]
	*Staphylococcus epidermidis*			
	*Staphylococcus saprophyticus*			

### Class 2 integron

Similar to the organization of class 1 integron, class 2 integron is commonly found to be associated with the Tn7 transposon family (Tn7 and its derivatives, such as Tn*1825*, Tn*1826* and Tn*4132*), carrying both of its recombination site *attI2* and promoter Pc found within such transposons [19]. Its 3′ conserved segment (3′-CS) contains 5 tns genes (*tnsA*, *tnsB*, *tnsC*, *tnsD* and *tnsE*) functioning in the movements of transposon [56], which mediates the mobility of class 2 integron via a preferential insertion into a unique site within bacterial chromosomes [30, 57]. The homology of amino-acid sequences of a typical *intI2* gene are found to be less than 50 % comparing to the *intI1*, and unfunctional due to the replacement of the internal termination codon with glutamic acid (amino acid 179) and thus the production of a shorter and inactive polypeptide which was unable to catalyse the recombination reaction [58]. Though the origin of this stop codon still remains unclear, the two current explanations for this potentially pseudogene are available as follows: (1) the regulatory function; (2) the functioning from presence of other type of integrase (mostly *intI1*). Such assumption has been supported by the simultaneous carriage of class 1 and class 2 integrons, the limited number of different arrays of gene cassettes, as well as the low diversity of cassette genes obtained. Despite its capability of site specific excision and integration of gene cassettes precisely into *attI2*, *intI2* is unable to recognize the *attC* sites of gene cassettes from class 1 integrons and mediate further integration. However, class 2 integrons share identical gene cassettes with class 1 integrons, such as *dfrA1*, *sat1* and *aadA1*. The classic structure of class 2 integrons contain an array of gene cassettes, including dihydrofolate reductase (*dfrA1*), streptothricin acetyltransferase (*sat1*), and aminoglycoside adenyltransferase (*aadA1*), which confer resistance to trimethoprim, streptothricin and streptomycin/spectinomycin, respectively [19, 57]. However, in the past decade, novel rearrangements of cassettes and resistance genes had been reported and identified. In detail, an erythromycin esterase gene (*ere*A) was detected in a class 2 integron containing its own promoter and capable of being propagated by a class 2 integron with an insertion sequence element (IS*1*) upstream of the *intI2* gene [59]. Also, a novel rearrangement of a class 2 integron (Tn*7*::In*2*-*8*) with new cassettes in the variable region were recovered from 3 *Acinetobacter baumannii* isolates and its structure contained 6 antibiotic resistance genes within the variable region (3 additional genes *sat2*, *aadB* and *catB2* inserted upstream of the 3 conventional antibiotic resistance genes of Tn*7* class 2 integron, as indicated in Table 3) [60]. In addition, the novel cassette arrays of class 2 integron (Tn*7*::In*2*-*1*) was found in *B. cenocepacia* strain and an unusual array (*sat*-*sat1*-*aadA1*) in *S. enteritidis* (Table 3) [16]. The mechanism and evolution of such novel cassette arrays require further investigation and surveillance. Considered to be a major contributor to the wide spread and distribution of antibiotic resistance in microorganisms, class 2 integrons have been commonly reported in some species of Gram-negative organisms such as *Acinetobacter*, *Enterobacteriaceae*, *Salmonella* and *Psuedomonas*, with a low occurrence and prevalence comparing with class 1 integron (Table 4) [16, 17, 61–66]. From a retrospective integrons surveillance conducted in Guangzhou China during 2001–2005, class 2 integron had also been occasionally detected (5.7 %, 33/583) of all tested isolates, with species of bacteria including *P. aeruginosa*, *E.coli*, *E. faecalis*, *Proteus vulgaris* and *Proteus mirabilis* strains, and cassettes arrays *dfrA1*-*sat1*-*aadA1* obtained for all strains [19, 52].

**Table 3:**
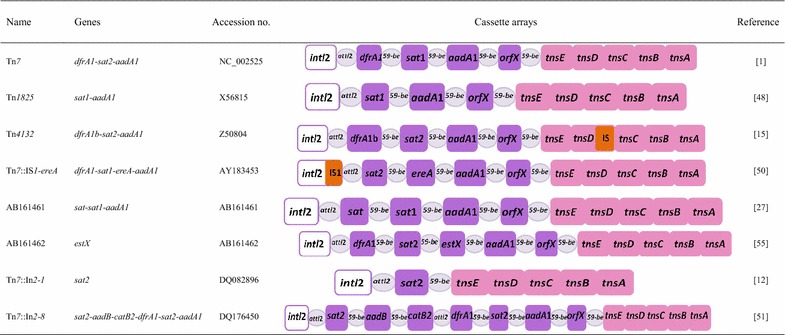
Summary of different structures of class 2 integrons reported in previous studies

**Table 4:** Occurrence and prevalence of class 2, 3, and 4 integrons in Gram-positive and Gram-negative bacteria

Bacterial	Occurrence of integrons and the array of gene cassettes	Sampling	Reference
Class 2 integrons			
* Escherichia coli*	7.4 % (31/417); *dfrA1-sat2-aadA1* (77.4 %, 24/31), *estX-sat2-aadA1* (19.4 %, 6/31) and *estX-sat2-△aadA1* (3.2 %, 1/31)	BfT-GermVet monitoring study, Germany, 2004–2006	[67]
* Enterobacteriaceae*	34.9 % (52/149); II2 (Tn*7*), III2 (*estX-sat2-aadA1-orfX*, most widely distributed) and IV2 (*aadA1*, first reported)	*E. coli* amd *K. pneumoniae* strains from swine and chickens, Portugal	[62]
* E.coli*	3.0 % (3/100)	Spain	[65]
* E. coli*	3.6 % (4/111); *dfrA1-sat1-aadA1*	Preliminary study, Guangzhou, China	[68]
* E. coli*	One out of 322	Irrigation water and associated sediments, El Paso, Presidio and Weslaco	[69]
Coliforms	2.7 % (5/183)	Rivers in northern region of Turkey	[63]
* Pseudomona* *aeruginosa*	19.5 % (23/118); *dfrA1-sat1-aadA1*, first report of class 2 integron in this species of bacteria	Preliminary study, Guangzhou, China	[19]
* Shigella flexneri*	100 % (58/58); *dfrA1-sat1-aadA1*	Stool samples of sporadic diarrheic patients, China, 2005–2006	[70]
* S. sonnei*i	93 % (2/43)	Adult patients with diarrhoea, Senegal	[71]
* S. enterica*	85 contemporary multi-drug resistant D-Tartrate-Positive isolates; *dfrA1*-*sat1*-*aadA1*	*S. enterica* Serovar Paratyphi B isolates Germany, 1995–2001	[72]
* S. enteritidis*	4.3 %; *estX-sat2-aadA1*	Poultry samples, Japan	[33]
* E. faecalis*	Two strains harboring Class 1 and 2 integrons; *dfrA1-sat1-aadA1*, first evidence of class 2 integron in G^+^ bacteria	Preliminary study, Guangzhou, China	[52]
Class 3 integrons			
* E.coli*		Australia	[73]
* E.coli*	*ges1*/*oxa10:aac(6′)*	Switzerland	[74]
* Serratia marcescens*	*imp1*/*aacA4*	Japan	[75]
* Klebsiella pneumoniae*	*ges1*/*oxa10*:*aacA4*	The urine of an intensive care unit patient in Portugal	[76]
Class 4 integrons			
* Vibrio cholerae*		Collection de I’Institut Pasteur (CIP)	[77, 78]
* V. metschnikovii*			[77]

### Class 3 integron

Comparing with class 2 integron, class 3 integron contains a similar structure, as both *IntI1* and *IntI3* are part of the soil/freshwater proteobacteria group, with *IntI2* found among the marine γ-proteobacteria group. Sharing similar function with *IntI1*, *IntI3* has been identified to be capable of both catalyzing excision of integrated cassettes and integration of circularized cassettes into the *attI3* site with a significantly lower recombination frequencies occurred between a 59-be and secondary sites than that observed with *IntI1*, and integrating various cassettes containing different *attC* sites into the *attI3* site which was localized to a short region adjacent to *intI3* [73]. This class of integron was firstly identificated from *Serratia marcescens* isolates in Japan in 1993, and then found to be associated with *bla*GES-1 from *Klebsiella pneumoniae* strain FFUL 22K. Its identification has been limited within a few microorganisms including *Acinetobacter* spp., *Alcaligenes*, *Citrobacter freundii*, *Escherichia coli*, *Klebsiella pneumoniae*, *Pseudomonas aeruginosa*, *Pseudomonas putida*, *Salmonella* spp and *Serratia marcescens* [73, 76, 79, 80] and mostly reported in low occurrence with common association with mediation IMP-1 metallo-beta-lactamase [73]. However, a class 3 integron had been lately identified containing *bla*GES-1 within the IncQ plasmid from *E. coli* [74]. The occurrence and identification rate of class 3 integron has been ranged from 0 to 10 %, with reports including a surveillance of 587 Gram-negative bacteria demonstrating high-level resistance to both ceftazidime and sulbactam-cefoperazone, with 0.7 % (4/587) isolates harboring class 3 integron and an occasional report with an occurrence of 7 % of veterinary isolates positive for class 3 integrase by DNA–DNA hybridization, despite discrepency when confirmed by PCR [80].

### Class 4 integron

Harboring a large array of gene cassettes encoding adaptations with extension beyond antibiotic resistance and pathogenicity, class 4 integron had been firstly detected in *Vibrio* isolates, with its existence pre-dating the antibiotic era [77]. This distinctive class of integron had been distinguished from other RIs by two key features including both the incorporated hundreds of cassettes (For *V. cholerae*, at least 216 unidentified genes in an array of 179 cassettes had been identified from the cluster of VCR-associated ORFs, occupying approximate 3 % of the genome) and the high homology between the *attC* sites of those gathered cassettes [78]. Despite its unique array of cassettes, identification of class 4 integron has been limited within microorganisms such as the *Vibrionaceae*, *Shewanella*, *Xanthomonas*, *Pseudomonad*, and other proteobacteria [20, 78, 81]. To date, class 4 integrons have been found to carry gene cassettes imparting resistance to the antibiotics chloramphenicol and fosfomycin [12].

## Novel perspectives in integrons

### Integrons in food borne bacteria

Remaining as one of the leading concerns in public health and food safety, food-borne infections and diseases have been reported to be caused by a large variety of pathogens that contaminate food and food products. Major food borne pathogens include *S. aureus*, *E. coli* O157, *V. parahaemolyticus*, *Salmonella* spp. and *L. monocytogenes*, which are responsible for 14 million illnesses, 60,000 hospitalizations and 1800 deaths annually [19, 46–48, 82–88]. Lately, indiscriminate abuse of existing antibiotics in veterinary treatment for a wide range of infectious diseases caused by bacteria in animals is found to be common, and food borne pathogens have been commonly identified from food poisoning, contamination of various food samples such as milk, pork, chicken, veal, beef, turkey and lamb meat, as well as in food production animals such as cattle, chickens, pigs and cows. As antibiotic resistant food borne pathogens have been considered to be a major contributor to both health-care associated and food-borne illnesses, carriage of such bacteria in a wide variety of food and food production animals are no longer limited solely to food hazard, but also poses a significant occupational risk for the industrial staff, such as handlers, asymptomatic carriers and uncolonized individuals [87]. According to our preliminary surveillance of antimicrobial resistance conducted on 96 food borne strains (including 32 *Salmonella* spp., 32 *E. coli* and 32 *S. aureus*), the phenotypic correlation existed among the aspects of antibiotic susceptibility, class 1 integrons and the abilities of biofilm formation had been firstly studied (data unpublished). In addition, class 1 integron had been discovered from food borne *S. aureus* strain, representing the first evidence of class 1 integron from food borne Gram-positive microorganisms as *Staphylococcus*. This novel finding may offer significant guidance in effective control on dissemination of antibiotic resistance of foodborne pathogens, nevertheless, the occurrence and prevalence of integrons, including class 1 integron and other classes of integrons, as well as the role of such integrons play in the antimicrobial resistance in food safety, require further investigation.

## Concluding remarks

Antimicrobial resistance still remains the leading concern in global public health and food safety, as bacteria are capable of obtaining resistance gene through either genetic mutation or horizontal transfer of resistance genes. Horizontal transfer of resistance genes are considered to be the major cause to facilitate the rapid spread of antibiotic resistance in microbes. As a frequently reported resistance mechanism served as horizontal transfer among microbes and found to be a common genetic element existed in 9 % of bacteria and representatives from a broad range of phyla and environments, integrons play core role in antibiotic resistance of microorganisms and have been shown to contribute to the wide spread and distribution of antibiotic resistant genes among bacteria, as well as the bacterial evolution and adaption [89]. The currently available studies and investigations have been restricted and limited within class 1 integron with perspectives on Gram-negative bacteria. Nevertheless, class 1 integron on Gram-positive microorganisms, together with class 2, 3 and 4 integrons has barely been touched upon, making such concerns potentially be unnoticed and neglected antibiotic resistance determinants. As consequence, identification of integrons regarding the species of involved microorganisms, occurrence and prevalence of different classes of integrons in certain species of bacteria, distribution and spread of integrons and cassettes arrays, as well as the role of such integrons play in the dissemination and spread of antimicrobial resistance, require further investigation.
